# Mitochondrial Genomes of Four Millipedes (Diplopoda: Spirostreptida and Spirobolida) Unveil Phylogenetic Novelty and Gene Rearrangement Patterns

**DOI:** 10.3390/cimb47060476

**Published:** 2025-06-19

**Authors:** Yingzhu Li, Gaoji Zhang, Wei Xu, Tangjun Xu, Lingna Li, Ming Gao, Jiachen Wang, Hongyi Liu

**Affiliations:** College of Life Sciences, Nanjing Forestry University, Nanjing 210037, China; 2362070368@njfu.edu.cn (Y.L.); zhanggaoji@njfu.edu.cn (G.Z.); xuwei2001@njfu.edu.cn (W.X.); xutangjun@njfu.edu.cn (T.X.); 8241811887@njfu.edu.cn (L.L.); 18934832876@163.com (M.G.); wangjiachen@njfu.edu.cn (J.W.)

**Keywords:** millipedes, mitochondrial genomes, phylogenetic analysis, Spirostereptida, Spirobolida

## Abstract

Millipedes (Diplopoda) are crucial decomposers in soil ecosystems, as they play a vital role in organic matter degradation while also holding potential as bioindicators of environmental health. This study deciphered the complete mitogenomes of four millipede species (Diplopoda: Spirostreptida and Spirobolida) using next-generation sequencing technology, thus revealing evolutionary relationships among diplopod taxa and characterizing mitochondrial genomic features. The full mitochondrial sequences of *Agaricogonopus acrotrifoliolatus*, *Bilingulus sinicus*, *Paraspirobolus lucifugus*, and *Trigoniulus corallinus*, ranged in size from 14,906 to 15,879 bp, with each containing 37 typical genes and one D-loop region. Notably, the D-loop regions of *A. acrotrifoliolatus* and *B. sinicus* were positioned atypically, thus indicating structural rearrangements. A nucleotide composition analysis revealed pronounced AT-skews, with tRNA sequences exhibiting the highest A+T content. Ka/Ks ratios demonstrated that the ND5 gene experienced the weakest purifying selection pressure, thus suggesting its potential role in adaptive evolution. The results of the phylogenetic analysis showed genetic relationships between the three orders of ((Julida, Spirostreptida), Spirobolida), which was inconsistent with the previous conclusion regarding the three orders, obtained through morphological studies: ((Julida, Spirobolida), Spirostreptida). These findings highlight the role of the mitochondrial genome in resolving phylogenetic conflicts and provide important insights for further studies on millipedes.

## 1. Introduction

Millipedes represent a diverse group of soil arthropods and are classified under the class Diplopoda (phylum Arthropoda, subphylum Myriapoda). In most ecological environments, millipedes are regarded as general decomposers [1] and directly participate in the decomposition of soil and litter (such as fallen leaves) while also playing a crucial role in maintaining soil fertility [2]. Millipedes are key in the calcium cycle and affect the mineral balance of soil [3]. They can effectively increase the biomass of fungi and bacteria and promote an increase in the activities of phenoloxidase and glucosidase [4]. In addition, various activities of millipedes have a positive promoting effect on maintaining the population size within the algal community and preserving the richness of soil algal species [5]. Based on the taxonomic framework established in 2011, Diplopoda comprises approximately 12,000 described species systematically organized into 3 subclasses and 26 orders [6]. However, molecular and biogeographic evidence indicates the existence of many undiscovered new species, with a conservative estimate of 15,000–20,000 extant species [6], while some studies have proposed higher estimates of up to 80,000 [7]. Although this class has important ecological functions and many members, little is known about the diversity, morphology, and phylogenetics of this group.

Most of the previous phylogenetic analyses were based on morphology. However, due to the high species diversity of millipedes, the morphological characteristics of traditional taxonomic dependence are vulnerable to the influence of environmental adaptation [8]. In addition, the morphological classification of millipedes often depends on the characteristics of male gonopods, but many groups of male specimens are scarce [9]. The emergence of molecular technology allows for a new perspective in terms of species identification and phylogenetic analysis. Mitochondria are important organelles in cells and are found in most eukaryotic cells. They possess mitochondrial DNA, which has a closed, circular, double-stranded structure and replicates semi-conservatively [10]. The mitochondrial genome of an arthropod is a small extrachromosomal genome with a simple and conserved structure and a length of about 16 kb. The genome contains 13 protein-coding genes (PCGs), including COI, COII, ATP8, ATP6, COX3, ND3, ND5, ND4, ND4L, ND6, Cytb, ND1, and ND2, as well as 2 ribosomal RNA genes (16S and 12S), 22 tRNA genes, and a non-coding D-loop region [11]. The mitogenome has become an important molecular tool for molecular systematic research and species identification due to its unique biological characteristics. This genome has shown significant advantages in the research fields of analyzing species formation mechanisms, reconstructing evolutionary relationships, and revealing population genetic structures [12,13]. In some studies, mitogenomes have been used to perform phylogenetic analyses of species of the orders Spirobolida and Spirostreptida [14,15,16]. However, the progress of research on the mitogenome of millipedes is relatively slow, and only around 30 complete mitochondrial sequences of millipedes have been published on NCBI. Due to the limited number of known mitogenome sequences, our understanding of the phylogeny and genetic evolution of millipedes is greatly hindered. Moreover, published cladistic or phylogenetic studies are relatively scarce and fail to provide convincing evidence [17].

In view of the importance and lagging research status of millipedes in the ecosystem and to better study their phylogenetic relationship, four species of millipedes were selected in this study, namely *Agaricogonopus acrotrifoliatus* (Zhang, 1997), *Bilingulus sinicus* (Zhang & Li, 1981), *Paraspirobolus lucius* (Gervais, 1836), and *Trigoniulus corallinus* (Eydoux & Souleyet, 1842). We assembled and characterized the mitogenomes of four species and then analyzed these mitogenomes by evaluating genome size, nucleotide composition, codon usage, AT-skews, and the Ka/Ks value of PCGs. To fill the gap in the study of millipede phylogeny, the theoretical framework of arthropod evolution was optimized. Based on the whole mitogenome of millipedes, a phylogenetic tree of millipedes was constructed using the Bayesian inference (BI) and maximum likelihood (ML) methods. The results of this study will provide new molecular evidence for the taxonomic study of millipedes and contribute to the study of the arthropod mitogenome.

## 2. Materials and Methods

### 2.1. Sample Collection and DNA Extraction

Four millipedes distributed across distinct biogeographic regions of China were collected for this study: *A. acrotrifoliolatus* (Xishuangbanna Dai Autonomous Prefecture, Yunnan Province, 22° 00′ N 100° 47′ E), *B. sinicus* (Sanming City, Fujian Province, 26° 16′ N 117° 38′ E), *P. lucifugus* (Hezhou City, Guangxi Zhuang Autonomous Region, 24° 40′ N 111° 55′ E), and *T. corallinus* (Zhanjiang City, Guangdong Province, 21° 27′ N 110° 35′ E). The identification of these specimens was based on the morphological criteria described in the literature [18,19,20,21]. Genomic DNA was extracted from a pair of amputated legs using DNAiso (Takara, Beijing, China). Four millipedes were subsequently raised at the Zoology Laboratory of Nanjing Forestry University. The extracted DNA was stored in a cryogenic freezer maintained at −80 °C in the Zoology Laboratory of Nanjing Forestry University.

### 2.2. Next-Generation Sequencing and Assembling

Library construction and sequencing were carried out by Novogene in Nanjing, China. The HiSeq 2500 platform from Illumina (Personal, Shanghai, China), was utilized, and the entire process was performed in strict accordance with the manufacturer’s protocol to generate 150 bp paired-end reads. To obtain high-quality data, the original sequencing data was further filtered. Data filtering was conducted as follows: (1) Contaminated sequences were removed from the 3′-end of the adapter. (2) The sliding window method was used for quality filtering. The window size was set to 9 bp and the step size to 1 bp. One base was moved forward at a time, and the average quality score (Q-score) of the window was calculated using nine bases. If the average Q-score of the window was ≤20, only the second base from the reciprocal of the window and the previous base were retained. (3) Reads containing fewer than five identifiable bases were discarded.

We utilized two tools to assemble the mitogenome. De novo assembly refers to the method of splicing sequencing reads into a complete genome directly using an algorithm, without relying on known sequence information; the reference sequence assembly is a strategy to map the reads to the reference sequence and fill the gap based on the existing reference genome. For *A. acrotrifoliolatus* and *B. sinicus*, due to the lack of closely related and annotated mitochondrial genomes in public databases (such as GenBank), reference sequence assembly could not be implemented. Both of these methods have inherent limitations and require manual correction after assembly is completed. Specifically, if there is no corresponding region in the reference genome for the target species, then the sequence of the target genome may be wrongly identified as a sequencing gap, and if the de novo assembly fails to correctly interpret repetitive sequences, it may lead to errors in the calculation of the length of a certain region. The mitogenomes of *A. acrotrifoliolatus* and *B. sinicus* were assembled from scratch by utilizing the mitogenome toolkit MitoZ 3.0 [22]. In Geneious Prime v2024.0.7, complete mitogenomes were assembled using high-quality reads, with *Litostrophus scaber* (Verhoeff, 1938) and *Spirobolus bungii* (Brandt, 1833) being used as reference templates to assemble the mitogenomes of *P. lucifugus* and *T. corallinus*, respectively. The medium-sensitivity/speed option was utilized for the assembly. Consensus sequences were generated using a 50% base call threshold, thereby obtaining the complete mitogenomes. The terminal regions of the final assembly were examined manually to achieve alignment and form a circular mitogenome.

### 2.3. Annotation and Sequence Analysis

The preliminary examination of the four mitogenomes was carried out using Seqman v7.1.0 and the MITOS Web Server for sequence alignment and gene identification [23,24]. The MITOS Web Server was utilized to locate tRNAs, rRNAs, and D-loops. PCGs were predicted using both MITOS WebServer (http://mitos.bioinf.uni-leipzig.de/index.py) and the BLAST v2.16.0 tool on the NCBI website. The correct mitogenomes were submitted to GenBank (accession numbers: PQ602473 for *A*. *acrotrifoliolatus*, PQ568882 for *B*. *sinicus*, PQ625794 for *P*. *lucifugus*, and PQ459337 for *T*. *corallinus*). The gene maps of the mitogenomes were constructed utilizing the web application GeSeq [25]. Relative synonymous codon usage (RSCU) was computed using MEGA11, and the resulting image was visualized using PhyloSuite v1.2.3 [26,27]. Nucleotide compositional skewness was calculated according to the following formulae: AT-skew = (A − T)/(A + T) and GC-skew = (G − C)/(G + C) [28]. Additionally, the synonymous replacement rate (Ks), nonsynonymous replacement rate (Ka), and the ratio of the nonsynonymous replacement rate to the synonymous replacement rate (Ka/Ks) were determined through analysis in MEGA11 [29].

### 2.4. Phylogenetic Analysis

A total of 31 species of millipedes with complete mitogenomes, representing 11 families and 7 orders, were included in the phylogenetic analyses (Table 1).

*Cermatobius longicornis* (Takakuwa, 1939) was selected as the outgroup. A phylogenetic analysis of each dataset was performed using the PhyloSuite platform v1.2.3 through the BI and ML methods. The concatenated nucleotide sequences of the complete set of genes, which include 13 PCGs and 2 rRNA genes, were used for phylogenetic analysis. Multi-sequence alignment was first executed utilizing MAFFT v7.313, thereby rendering all sequences of equal length [30]. ModelFinder v2.2.0 was then used to select the optimal partitioning strategy and evolutionary model for the dataset [31]. The AIC (Akaike information criterion) and BIC (Bayesian information criterion) are two commonly used model selection criteria. Unlike the AIC, the BIC considers the number of samples. When the number of samples is too large, the BIC can effectively prevent the excessive model complexity caused by excessive model precision [32]. The results of the best-fit model are shown in Table 2.

There are two main methods used for constructing trees, namely the ML and BI methods. IQ-TREE performed the best in the dataset of multigene concatenation and obtained the maximum likelihood value. The construction of the phylogenetic tree was performed using the ML method, and the bootstrap value was set to 1000 repetitions. The construction of the BI tree requires determination of whether it converges. Generally, when the ASDSF value is lower than 0.01, the BI analysis results can be considered to have converged [33]. The interactive Tree of Life (iTOL) website was used to view and edit phylogenetic trees (https://itol.embl.de/ accessed on 18 December 2024).

## 3. Results and Discussion

### 3.1. Mitochondrial Genome Organization

**Figure 1:**
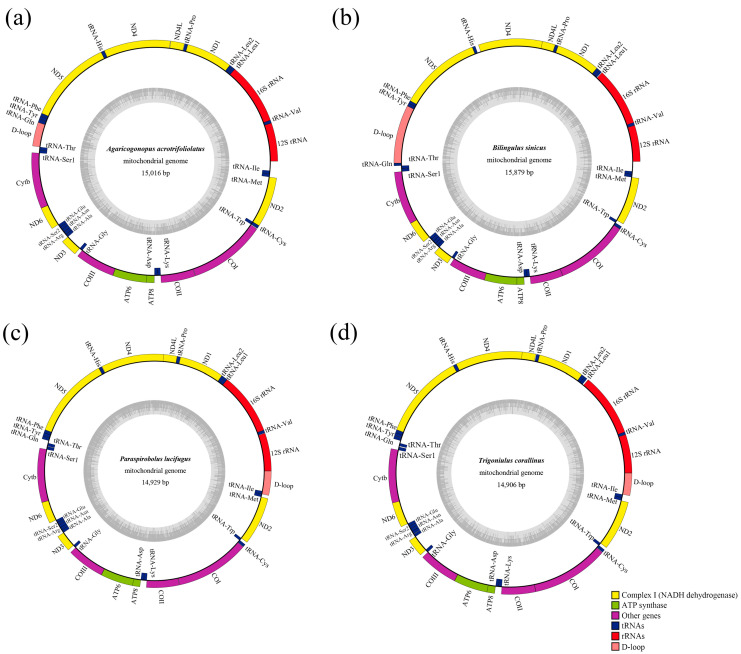
The mitogenomes of the four millipedes sequenced in this study: (**a**) *A. acrotrifoliolatus;* (**b**) *B. sinicus;* (**c**) *P. lucifugus;* (**d**) *T. corallinus*.

All four millipedes had complete mitogenomes, which consisted of circular and double-stranded molecules. The sizes of the four mitogenomes were 15,016 bp, 15,879 bp, 14,929 bp, and 14,906 bp (Figure 1). The mitogenomes included 37 typical mitochondrial genes (consisting of 13 PCGs, 22 tRNAs, and 2 rRNAs) and one D-loop region. Among these species, *T*. *corallinus* had the smallest mitogenome, whereas *B*. *sinicus* had the largest. Nine tRNAs and four PCGs were encoded on the minor strand, whereas the other genes were located on the major strand (Table 3). The phenomenon of gene overlap occurs in the mitogenomes of each species. Additionally, the longest gene overlap, which is 64 bp in length, was found between tRNA-Cys and tRNA-Trp in the mitogenome of *A*. *acrotrifoliolatus* (Table 3).

**Figure 2:**
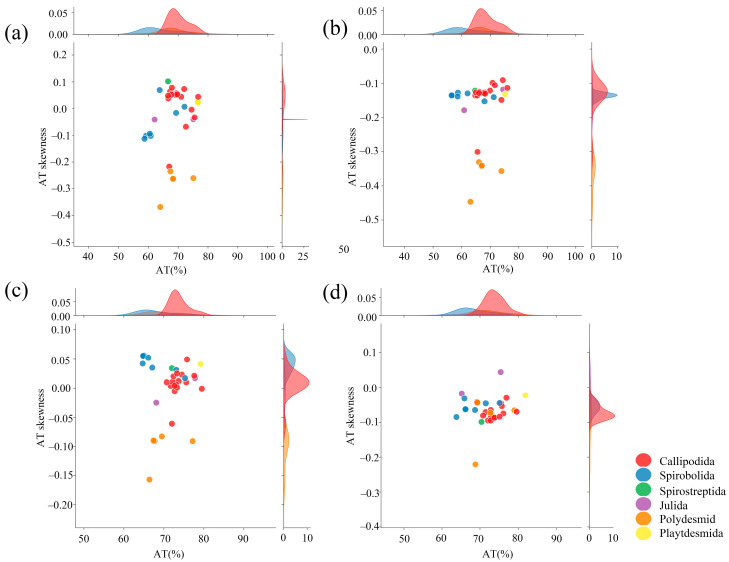
A+T content vs. AT-skew in the six orders of millipedes with complete mitogenomes. (**a**) Total genome; (**b**) PCGs; (**c**) tRNAs; (**d**) rRNAs. The ordinate represents AT-skew and the abscissa represents A+T content.

The analysis of the nucleotide composition within four complete mitogenomes entailed the calculation of the A+T content, AT-skew, and GC-skew (Table 4). The nucleotide composition of the four millipedes showed a preference for AT nucleotides, with a percentage ranging from 60.50% to 75.43%. The A+T contents of *Narceus annularus* (Rafinesque, 1820) (Spirobolida) and *Thyropygus allevatus* (Attems, 1950) (Spirostreptida) were 63.7 and 67.8%. Thus, this pattern is consistent with that observed in other millipedes [34,35]. Specifically, the tRNA sequences displayed a higher A+T content (64.68~77.24%), while the A+T content in PCGs, tRNAs, and D-loops was comparable to that of the complete mitogenome. A total of 31 species of millipedes with complete mitogenomes, belonging to 6 orders and 15 families, were included in the analysis of A+T content and AT-skew (Table 1 and Figure 2). The 31 millipede mitogenomes exhibited a comparable nucleotide composition. The AT-skews were slightly negative (−0.130~−0.003) and the GC-skews were positive (0.023~0.441) across the four mitogenomes (Table 4). The A+T content was consistently higher than the G+C content in various components, including the total genome (58.7~76.7%), PCGs (56.5~76.0%), tRNAs (64.7~79.5%), and rRNAs (63.8~81.9%). The AT-skews were almost positive, suggesting a higher frequency of adenine (A) than thymine (T).

### 3.2. Protein-Coding Genes and Codon Usage

The total sizes of the 13 PCGs in the four millipedes were 10,971 bp, 11,022 bp, 10,971 bp, and 11,007 bp, which accounted for 71.58%, 74.31%, 58.54%, and 71.21% of the complete mitogenomes, respectively. The A+T content of PCGs ranged from 58.54% to 74.31% of the 13 PCGs present in these four mitogenomes, and ATP8 had the smallest size, whereas ND5 was the largest.

In the four species of millipedes, the PCGs Cytb, COIII, ATP6, and COII are highly conserved, and their start codons are all ATG. Meanwhile, most PCGs start with the codon ATN (ATT/A/C). However, some PCGs are unstable, such as ND5 and COI, which start with special start codons GTG, TTA, ACG, and CGA. Four species were found to possess four types of termination codons, namely TAA, TAG, TA, and T. Seven protein-coding genes use the typical termination codons TAG (COI, COII, ND4L, ND4, and ND5) and TAA (ATP8 and ND1). However, some PCGs were terminated with special termination codons: single T and TA (Table 5). These special termination codons can also be found in other arthropods [36,37], and it is possible that these codons could be transformed into TAA or TAG to perform their formal functions [38].

**Figure 3:**
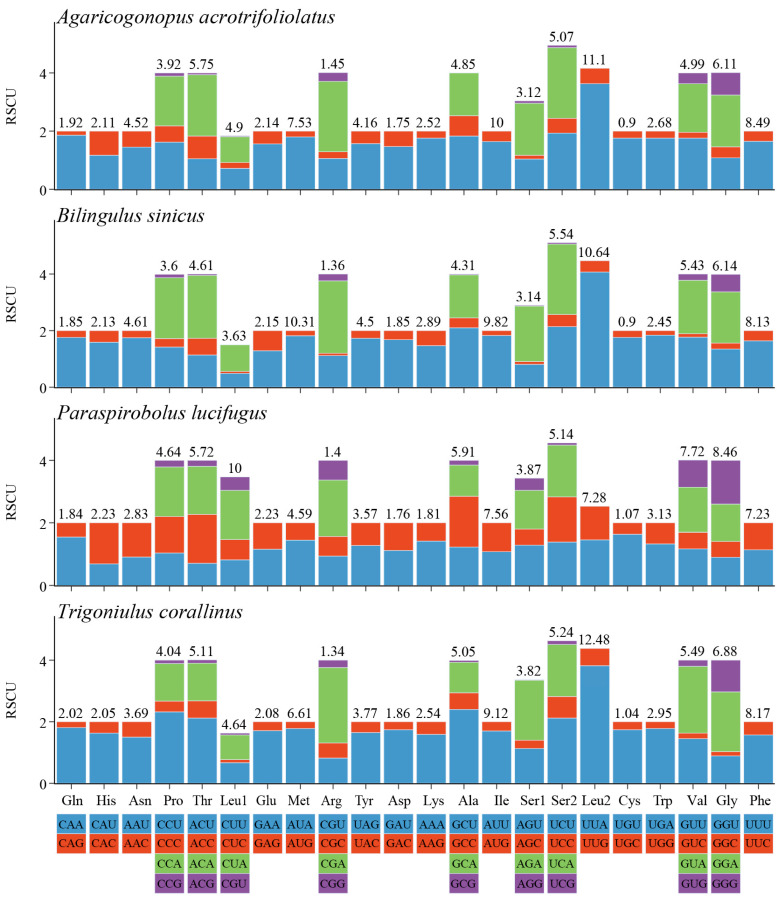
RSCU values of four species of millipedes; termination codon is not included.

The RSCU values of the four millipede species from Spirobolida and Spirostreptida were summarized in order to determine the frequency of synonymous codon usage (Figure 3). The RSCU of the four mitogenomes was similar. The three most frequently used amino acids were Leu2, Gly, and Val, whereas the three least frequently used codon families were Cys, Arg, and Gln. Similarly, the biased utilization of A+T nucleotides was manifested in the codon frequencies. The usage of codons ending in A/U was considerably higher than that of codons ending in C/G, which reflects the strong AT-skew of the third codon position.

**Figure 4:**
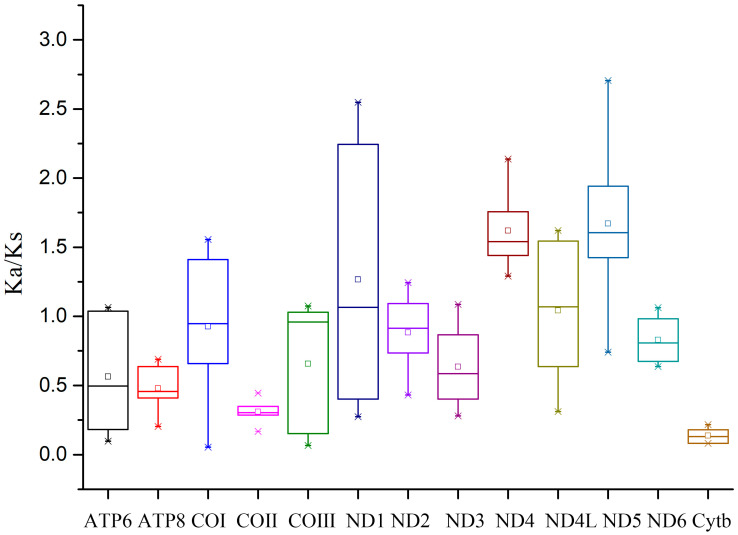
Ka/Ks values for the 13 PCGs of four millipede mitogenomes.

To measure the selection pressure, the ratio of the nonsynonymous substitution rate to the synonymous substitution rate (Ka/Ks) was computed for the 13 PCGs in the mitogenomes of the four species (Figure 4). Interestingly, the ND5 gene had the largest Ka/Ks value, thus suggesting that it faces the lowest selection pressure and evolves faster than the other PCGs in the mitogenomes [39]. Conversely, the Cytb gene exhibited the lowest average Ka/Ks value, thus indicating a slower evolutionary pace, possibly due to significant selection pressures [40]. Most PCGs showed Ka/Ks values below 1, thus suggesting a prevalence of purifying selection in the determination of the evolutionary trajectory of mitochondrial PCGs [41].

### 3.3. tRNAs, rRNAs, and D-Loops

There were twenty-two tRNAs in each of the four mitogenomes, with nine being from the major strand and thirteen transcribed from the minor strand (Table 2). The total sizes of the tRNAs were 1408 bp, 1385 bp, 1415 bp, and 1402 bp, which accounted for 9.38%, 8.72%, 9.48%, and 9.41%, respectively. The sizes of these tRNAs ranged from 55 bp (tRNA-Val in *T*. *corallinus*) to 69 bp (tRNA-Thr and tRNA-Ser1 in *P*. *lucifugus*).

In the four mitogenomes examined, it was found that the regions of the 12S rRNA and 16S rRNA containing rRNA in *A*. *acrotrifoliolatus* and *B*. *sinicus* were located between tRNA-Leu1 and tRNA-Ile, with tRNA-Val serving as a separator. The regions of the 12S rRNA and 16S rRNA of *P*. *lucifugus* and *T*. *corallinus* were located between tRNA-Leu1 and the D-loop and were also separated by tRNA-Val (Figure 1). The total length of 12S rRNA ranged from 758 bp (*B. sinicus*) to 784 bp (*P*. *lucifugus*), and that of 16S rRNA ranged from 810 bp (*B*. *sinicus*) to 850 bp (*P*. *lucifugus*).

**Figure 5:**
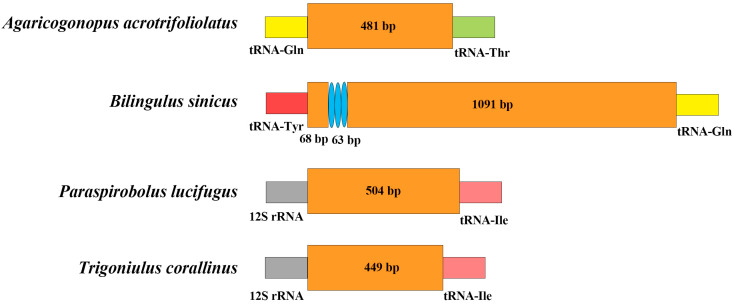
The structure of the control region of the mitogenomes of four kinds of millipedes. The blue ellipse indicates a tandem repeat sequence; the remaining areas are represented by orange boxes. Different tRNAs are distinguished by different colors.

The location of the D-loops is different in each order, and there are also differences within each order. In the four analyzed mitogenomes, the D-loops of *P. lucifugus* and *T. corallinus* (Spirobolida) were located between 12S rRNA and tRNA-Ile, whereas in *A. acrotrifoliolatus* and *B. sinicus* (Spirostreptida), the D-loops occupied an atypical position, thus indicating the structural rearrangement of the control region in the latter two species. The D-loop plays an important role in mtDNA gene transcription and mitochondrial replication, and its abnormal localization may affect mitochondrial gene expression, causing it to adapt to specific environmental pressures [42,43]. In addition, the D-loop usually evolves under evolutionary pressures, similarly to PCGs or mitogenomes [44]. In this study, the ND5 gene has the lowest Ka/Ks value, indicating that it is undergoing adaptive evolution, which may lead to the displacement of the D-loop region. Only the D-loop region of *B. sinicus* had repetitive sequences, and the 21 bp motif was copied three times. In addition, it was also found that the tRNA-Gln of *B. sinicus* had obvious positional changes (Figure 5). The sizes of the D-loops in the four mitogenomes ranged from 449 bp (*T*. *corallinus*) to 1222 bp (*B. sinicus*), thus accounting for 3.01% to 9.86%. The D-loop was the longest non-coding region, and the A+T content was higher, ranging from 67.57% to 78.47% (Table 4). These results are consistent with the characteristic high A+T content seen in the mitochondrial D-loops region of other invertebrates [45,46]. Since most invertebrate mitochondrial D-loops have high A+T content, the high A+T content observed here plays an important role in replication and transcription; therefore, the D-loops can be identified as putative [47].

### 3.4. Phylogenetic Analyses

**Figure 6:**
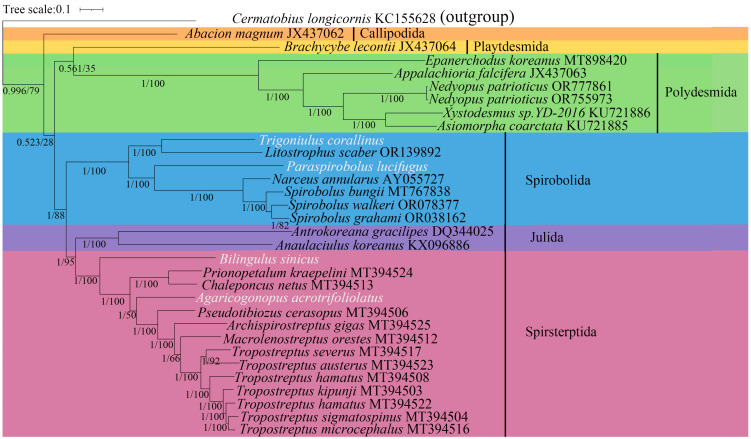
A phylogenetic tree was constructed for 31 species of Diplopoda, along with an outgroup (*C. longicornis*). This tree was based on thirteen PCGs and two rRNAs, and the BI and ML methods were employed. At the nodes of the tree, the numbers represent the statistical support values, with the values for BI shown as posterior probabilities and those for ML presented as bootstrap values. Notably, the four species that were newly determined in the current study are indicated in white.

We included 31 species of Diplopoda in the phylogenetic analyses and selected *C. longicornis* as an outgroup to root the phylogenetic trees using the BI and ML methods (Figure 6). From the phylogenetic tree, *A. acrotrifoliolatus* and *B. sinicus* (Spirostreptida) and *P. lucifugus* and *T. corallinus* (Spirobolida) were consistent with the gene arrangement pattern observed in Figure 6. In addition, a Julida was observed between Spirostreptida and Spirobolida. Thus, the mitogenome information found in this study likely suggests the following evolutionary relationship between the three orders: ((Julida, Spirostreptida), Spirobolida). Both morphological and mitochondrial phylogenetics demonstrated that Spirobolida, Julida, and Spirostreptida form sister groups, but there are discrepancies regarding the relationships between these three orders. Enghoff and Sierwald proposed the following phylogenetic relationships between the three orders through morphological studies: ((Julida, Spirobolida), Spirostreptida) [48,49]. Recent mitochondrial studies have shown that the relationship between the three orders is ((Julida, Spirostreptida), Spirobolida), which is consistent with the results of this study [50,51]. In conclusion, our research results differ from the morphological phylogenetic analysis. However, the crucial node formed by playtdesmida and polydesmida was particularly weakly supported, with a posterior probability of 0.561 and bootstrap support of 35%. The use of mitochondrial genomes alone, without independent corroboration with other molecules or morphological features, may lead to phylogenetic inferences that are not widely supported [52]. In phylogenetic studies of mitochondrial genomes in other arthropods, conflicts with morphological findings have also been observed. For example, the mitochondrial genomes of parasitic isopods exhibit extensive gene rearrangements, leading to significant phylogenetic conflicts with morphological characteristics [53]. Similar findings were observed in Arachnida; morphological analysis shows that the subfamily Ornithoctoninae is most closely related to Selenocosmiinae, while mitochondrial studies indicate that Theraphosinae has a closer genetic relationship with Ornithoctoninae [54]. The differences in mitochondrial and morphological phylogeny may be influenced by convergent evolution. Wesener showed that morphological adaptation evolved convergently in different and unrelated millipede orders and families [55]. Individual differences may also have an impact on the morphological classification of millipedes. In Juliformia, male gonopods often retract into the body, thus making it almost impossible to correctly assign a species of millipede to a recent taxon [8].

This study’s findings provide an important theoretical basis for further understanding the evolutionary history and biodiversity of millipedes, as well as the phylogeny of arthropods. However, our dataset only includes 31 species, which indicates that its coverage is very limited, and this may have an impact on the phylogenetic results. Mitochondrial genome analyses are often perturbed by horizontal gene transfer, pseudogenes, and gene introgression, phenomena that can yield spurious paraphyletic or polyphyletic groupings [52]. Expanding taxon sampling may enhance phylogenetic accuracy by diluting the impact of such aberrant signals. For example, when Zhang et al. conducted phylogenetic analysis using 24 millipede mitogenomes, the key node uniting Julida and Spirostreptida exhibited weak bootstrap support (65%), while the resolution among the three orders reached only 55% [51]. By contrast, our study increased support for these key nodes to 95% and 85%, respectively, demonstrating that enhanced sampling may strengthen the reliability of phylogenetic inferences. To resolve the existing taxonomic disputes and elucidate the higher-level phylogeny within millipede species, it would be beneficial to expand the sequencing work to cover more taxa.

## 4. Conclusions

In our study, we sequenced the complete mitochondrial genomes of four new species of centipedes. In *A. acrotrifoliolatus* and *B. sinicus*, an atypical D-loop translocation phenomenon was observed, and the evolutionary significance and potential mechanisms of this phenomenon were discussed. Through phylogenetic analysis, we found that the results were different from those of the morphological phylogenetic analysis, and we explored the possible reasons for this. Clarifying the differences between morphological phylogeny and molecular phylogeny is of great value in the field of evolutionary biology. This study fills an important gap in centipede molecular systematics and provides a foundation for future phylogenetic research. However, our dataset has limitations, as it covers only 31 millipede species. Future research should expand sequencing and include more taxa.

## Figures and Tables

**Table 1:** The mitogenomes used in phylogenetic analyses.

Class	Order	Family	Species	Length(bp)	Accession
Diplopoda	Callipodida	Callipodidae	*Abacion magnum* Loomis, 1943	15,160	JX437062.1
	Playtdesmida	Andrognathidae	*Brachycybe lecontii* Wood, 1864	15,115	JX437064.1
	polydesmida	Xystodesmidae	*Appalachioria falcifera* Keeton, 1959	15,282	JX437063.1
			*Xystodesmus sp. YD-2016* Cook, 1895	15,791	KU721886.1
		Paradoxosomatidae	*Asiomorpha coarctata* DeSaussure, 1860	15,644	KU721885.1
			*Nedyopu patrioticus* Attems, 1898	15,814	OR755973.1
		Polydesmidae	*Epanerchodus koreanus* Verhoeff, 1937	15,581	MT898420.1
			*Nedyopus patrioticus unicolor* Carl, 1902	15,798	OR777861.1
	Spirobolida	Trigoniulidae	*Trigoniulus corallinus* Gervais, 1847	14,906	PQ459337
		Pachybolidae	*Litostrophus scaber* Verhoeff, 1938	15,081	OR139892.1
		Spirobolellidae	*Paraspirobolus lucifugus* Gervais, 1836	14,929	PQ625794
		Spirobolidae	*Narceus annularus* Rafinesque, 1820	14,868	AY055727.1
			*Spirobolus bungii* Keeton, 1960	14,879	MT767838.1
			*Spirobolus grahami* Keeton, 1960	14,875	OR038162.1
			*Spirobolus walkeri* Pocock, 1895	14,879	OR078377.1
	Julida	Nemasomatidae	*Antrokoreana gracilipes* Verhoeff, 1938	14,747	DQ344025.1
		Julidae	*Anaulaciulus koreanus* Verhoeff, 1937	14,916	KX096886.1
	Spirostreptida	Cambalopsidae	*Bilingulus sinicus* Zhang & Li, 1981	15,879	PQ568882
		Odontopygidae	*Prionopetalum kraepelini* Attems, 1896	15,114	MT394524.1
			*Chaleponcus netus* Enghoff, 2014	15,093	MT394513.1
		Harpagophoridae	*Agaricogonopus acrotrifoliolatus* Zhang, 1997	15,016	PQ602473
		Spirostreptidae	*Pseudotibiozus cerasopus* Attems, 1914	15,121	MT394506.1
			*Archispirostreptus gigas* Peters, 1985	15,177	MT394525.1
			*Macrolenostreptus oreste* Hoffman & Howell, 1996	15,367	MT394512.1
			*Tropostreptus severus* Enghoff, 2017	15,209	MT394517.1
			*Tropostreptus austerus* Attems, 1950	15,261	MT394523.1
			*Tropostreptus hamatus* Demange, 1977	15,156	MT394508.1
			*Tropostreptus kipunji* Enghoff, 2017	15,170	MT394503.1
			*Tropostreptus droides* Enghoff, 2017	15,172	MT394522.1
			*Tropostreptus sigmatospinus* Enghoff, 2017	15,176	MT394504.1
			*Tropostreptus microcephalus* Enghoff, 2017	15,169	MT394516.1
Chilooda	Lithobiomorpha	Henicopidae	*Cermatobius longicornis* Takakuwa, 1939	16,833	KC155628.1

**Table 2:** Best-fit model of ML according to BIC and model of BI according to BIC.

Methods	Subset Partitions	Best Model
	Cytb+ATP6+COII+COIII	TIM2+F+I+G4
ML	ATP8	GTR+F+I+G4
	COI	
	ND2+ND3+ND6	
	12S rRNA+16S rRNA	
	ND1+ND4L+ND4+ND5	GTR+F+I+I+R5
BI	ATP8	GTR+F+I+G4
	COI	
	Cytb+ATP6+CO+COII	
	ND1+ND4L+ND4+ND5	
	ND2+ND3+ND6	
	Cytb+ATP6+COII+COIII	
	12S rRNA+16S rRNA	

**Table 3:** General features of the mitogenomes of *A*. *acrotrifoliolatus*, *B*. *sinicus*, *P*. *lucifugus*, and *T*. *corallinus*.

Gene	Location		Intergenic Nucleotides	Size (bp)
	From	To		
12S rRNA(+)	1/1/1/1	768/758/784/764	0/0/0/0	768/758/784/764
tRNA-Val(+)	769/760/789/766	829/821/849/820	0/1/4/1	61/62/61/55
16S rRNA(+)	826/810/850/816	2081/2045/2138/2080	−4/−12/0/−5	1256/1236/1289/1265
tRNA-Leu1(+)	2059/2058/2139/2107	2120/2118/2202/2170	−23/12/0/26	62/61/64/64
tRNA-Leu2(+)	2122/2120/2204/2171	2183/2184/2269/2234	1/1/1/0	62/65/66/64
ND1(+)	2184/2185/2270/2235	3102/3109/3193/3150	0/0/0/0	919/925/924/916
tRNA-Pro(+)	3103/3110/3198/3151	3163/3171/3264/3213	0/0/4/0	61/62/67/63
ND4L(+)	3169/3173/3256/3223	3441/3445/3546/3495	5/1/0/9	273/273/282/273
ND4(+)	3447/3469/3540/3489	4764/4788/4839/4820	5/23/−7/−7	1318/1320/1300/1332
tRNA-His(+)	4765/4848/4844/4820	4829/4909/4909/4884	0/59/4/−1	65/62/66/65
ND5(+)	4830/4910/4910/4885	6534/6629/6608/6588	0/0/0/0	1705/1720/1699/1704
tRNA-Phe(+)	6535/6630/6609/6589	6592/6687/6671/6649	0/0/0/0	58/58/63/61
tRNA-Tyr(+)	6539/6687/6670/6646	6657/6749/6735/6709	0/−1/−2/−4	65/63/66/64
tRNA-Gln(+)	6658/7972/6736/6711	6724/8027/6802/6775	0/0/0/1	67/56/67/65
tRNA-Thr(−)	7206/8028/6853/6825	7268/8090/6921/6887	0/0/50/49	63/63/69/63
tRNA-Ser1(−)	7269/8091/6928/6905	7334/8157/6996/6973	0/0/6/17	66/67/69/69
Cytb(−)	7335/8156/6995/6972	8453/9268/8113/8090	0/−2/−2/−2	1119/1113/1119/1119
ND6(−)	8454/9268/8114/8044	8910/9734/8564/8544	0/−1/0/−47	457/467/451/501
tRNA-Glu(−)	8914/9741/8565/8542	8977/9806/8629/8603	3/6/0/−3	64/66/65/62
tRNA-Ser2(−)	8978/9807/8630/8603	9037/9867/8687/8663	0/0/0/−1	60/61/58/61
tRNA-Asn(−)	9038/9867/8688/8664	9105/9929/8754/8728	0/−1/0/0	68/63/67/65
tRNA-Arg(−)	9106/9928/8754/8728	9172/9988/8815/8790	0/−2/−1/−1	67/61/62/63
tRNA-Ala(−)	9173/9987/8816/8794	9236/10,050/8883/8858	0/−2/0/3	64/64/68/65
ND3(−)	9235/10,050/8882/8857	9579/10,393/9232/9210	−2/−1/−2/−2	345/344/351/354
tRNA-Gly(−)	9580/10,394/9233/9205	9646/10,457/9295/9270	0/0/0/−6	67/64/63/66
COIII(−)	9646/10,456/9299/9271	10,431/11,241/10,076/10,048	−1/−2/3/0	786/786/778/778
ATP6(−)	10,432/11,228/10,076/10,049	11,107/11,911/10,752/10,724	0/−14/−1/0	676/684/677/676
ATP8(−)	11,101/11,905/10,746/10,718	11,256/12,060/10,901/10,873	−7/−7/−7/−7	156/156/156/156
tRNA-Asp(−)	11,257/12,061/10,902/10,874	11,319/12,126/10,965/10,937	0/0/0/0	63/66/64/64
tRNA-Lys(−)	11,320/12,126/10,966/10,937	11,387/12,190/11,031/11,003	0/−1/0/−1	68/65/66/67
COII(−)	11,388/12,192/11,032/11,004	12,069/12,872/11,707/11,682	0/1/0/0	682/681/676/679
COI(−)	12,073/12,873/11,711/11,683	13,605/14,411/13,243/13,213	3/0/3/0	1533/1539/1533/1531
tRNA-Cys(+)	13,616/14,412/13,248/13,219	13,680/14,473/13,310/13,281	10/0/4/5	65/62/63/63
tRNA-Trp(−)	13,617/14,474/13,303/13,274	13,681/14,536/13,364/13,336	−64/0/−8/−8	65/63/62/63
ND2(−)	13,680/14,535/13,365/13,335	14,687/15,536/14,361/14,327	−2/−2/0/−2	1008/1002/997/993
tRNA-Met(−)	14,688/15,537/14,362/14,328	14,755/15,601/14,425/14,393	0/0/0/0	68/65/64/66
tRNA-Ile(−)	14,756/15,601/14,425/14,393	14,821/15,666/14,491/14,457	0/−1/−1/−1	66/66/67/65
D-loop	6725/6750/14,426/14,458	7205/7971/14,929/14,906	0/0/0/0	481/1222/504/449

**Table 4:** The composition and skewness of the four mitogenomes.

Species	Region	Size (bp)	A+T %	AT-Skew	GC-Skew
*Agaricogonopus acrotrifoliolatus*	Whole genome	15,016	72.57	−0.068	0.342
	PCGs	10,971	71.58	−0.106	−0.066
	tRNAs	1408	75.81	0.049	0.079
	rRNAs	2024	75.10	−0.084	0.385
	D-loop	481	67.57	0.003	0.295
*Bilingulus sinicus*	Whole genome	15,879	75.43	0.033	0.323
	PCGs	11,022	74.31	0.091	0.025
	tRNAs	1385	77.24	0.021	0.129
	rRNAs	1994	75.68	−0.054	0.381
	D-loop	1222	78.47	0.051	0.353
*Paraspirobolus lucifugus*	Whole genome	15,016	60.50	−0.068	0.342
	PCGs	10,971	58.54	−0.106	−0.066
	tRNAs	1415	64.68	0.049	0.07
	rRNAs	2024	65.90	−0.084	0.385
	D-loop	504	68.65	0.069	0.265
*Trigoniulus corallinus*	Whole genome	14,906	72.05	0.007	0.317
	PCGs	11,007	71.21	−0.141	0.013
	tRNAs	1402	75.32	0.017	0.092
	rRNAs	2029	75.11	0.045	0.386
	D-loop	449	71.04	0.065	0.246

**Table 5:** Comparison of start and stop codons of mitochondrial protein-coding genes in four mitogenomes.

Gene	Codon	
	Start	Stop
ND1	ATG/ATT/ATT/ATA	TAA/T/TAA/T
ND4L	ATG/ATG/GTG/ATG	TAA/TAA/TAG/TAA
ND4	ATA/ATG/ATG/ATG	T/T/T/TAA
ND5	TTA/GTG/GTG/ATT	T/T/T/TAG
Cytb	ATG/ATG/ATG/ATG	TAA/TAG/TAA/TAA
ND6	ATA/ATT/ATC/ATA	T/TA/T/TAA
ND3	ATA/ATT/ATA/ATA	TAG/TA/TAG/TAG
COIII	ATG/ATG/ATG/ATG	TAG/TAA/T/T
ATP6	ATG/ATG/ATG/ATG	T/TAA/TA/T
ATP8	ATT/ATT/ATA/ATT	TAA/TAA/TAA/TAA
COII	ATG/ATG/ATG/ATG	T/TAG/T/T
COI	ACG/ATT/ACG/CGA	TAA/TAA/TAA/TTT
ND2	ATT/ATT/ATC/ATT	TAA/TAA/TAA/TAA

## Data Availability

DNA sequences: GenBank accession number PQ602473 for *A*. *acrotrifoliolatus*, PQ568882 for *B*. *sinicus*, PQ625794 for *P*. *lucifugus*, and PQ459337 for *T*. *corallinus*.
